# The Relationship Between Psychological Pain, Spiritual Well-Being, and Social Support in Turkish Women Undergoing Therapeutic or Elective Abortion

**DOI:** 10.1007/s10943-024-02087-4

**Published:** 2024-07-17

**Authors:** Figen Alp Yılmaz, Dilek Avci

**Affiliations:** 1 Faculty of Health Sciences, Alanya Alaaddin Keykubat University, Antalya, Turkey; 2 Faculty of Health Sciences, Bandirma Onyedi Eylul University, Balikesir, Turkey

**Keywords:** Abortion, Psychological pain, Spiritual well-being, Social support

## Abstract

This study was conducted to determine the relationship between psychological pain, spiritual well-being, and social support among Turkish women undergoing therapeutic or elective abortion. The cross-sectional study was conducted with 342 women who were hospitalized in the gynecology and obstetrics service of a city hospital between March 2021 and December 2022 in Turkey. The mean psychological pain, spiritual well-being and perceived social support scores of women undergoing therapeutic/elective abortion were 26.96 ± 11.21, 110.72 ± 13.09 and 64.09 ± 15.62, respectively. There were a significant negative correlation between psychological pain with spiritual well-being and social support. According to linear regression analysis, spiritual well-being, social support, age, employment status, economic level, history of abortion, number of abortion, current abortion type, and gestational week were statistically significant predictive factors of psychological pain. Therefore, healthcare providers can provide individualized psychosocial-spiritual care and counseling services that decrease psychological pain in women after therapeutic or elective abortion.

## Introduction

Pregnancy is a normal physiological process; however, some complications that may endanger the health of the mother and the fetus may develop during this period (Traylor et al., 2020). The leading one of these complications is abortion which is manifested by vaginal bleeding (Li et al., 2021). Abortion is defined as “the process of terminating the life of the embryo implanted in the uterus by a physician through various methods, either at the woman’s own request or with written consent due to medical necessity” (McKinney et al., 2018). About 10–15% of clinically diagnosed pregnancies and 60% of all pregnancies result in abortion (Sesay et al., 2023). While abortions can be seen as spontaneous abortion as a complication of pregnancy, there are also other forms, namely elective abortion, in which the pregnancy is terminated with the consent of the expectant mother and father, even though there is no complication, or therapeutic abortion, in which the pregnancy is compulsorily terminated in cases that would endanger the health of the mother and the fetus (Demirel et al., 2022). Ending pregnancy by medical termination due to fetal anomaly or maternal health risks is one of the most difficult and painful life experiences for parents (Sun et al., 2018).

Women who will get an abortion often experience symptoms, such as sadness, helplessness, guilt, and suffering (Herbert et al., 2022). Pregnancy loss differs from the loss of a person in several ways. When a person dies, a piece of the past is lost, causing mourning and pain for those who have had a place in the person’s life (Das et al., 2021). Conversely, when a baby is lost, part of the future is lost, and the family may experience psychological pain for everything this child will represent. The concept of psychological pain (psychache) was first coined by Shneidman (1993) and was defined as the process of mental suffering that may result from situations, such as experiencing loss, exposure to traumatic events, and failure to meet basic needs (Demirkol et al., 2018). It is thought that abortion, a traumatic life event, may also cause psychological pain in women (Crockett et al., 2021).

Spiritual beliefs and values are very important for women to cope with this experience (Alipanahpour et al., 2023). Spiritual well-being is defined as “the feeling of having meaning and purpose in life, being able to use one’s inner power/resources, believing in a higher power, and establishing a relationship with that power” (Gomez & Fisher, 2003). It is stated that spiritual well-being is a unique power that facilitates coping with stress and increases psychological well-being for women who have had an abortion (Eklund et al., 2022; Wright, 2020). In addition to spiritual well-being, social support is an important resource for individuals to cope with a traumatic event (Iwanowicz-Palus et al., 2021). Women who experience pregnancy loss need the support of those around them, but friends and family often have difficulty understanding the full extent of the loss. Lack of perceived support after pregnancy loss causes pathological grief, anxiety, depression, psychological distress, and post-traumatic stress disorder (Levy and Avitsur, 2022). It is reported that social support received from partners, family, or friends during this process is effective in reducing the emotional responses that women experience most intensely in the post-abortion period (Hendrix et al., 2023; Iwanowicz-Palus et al., 2021)

Spirituality and social support are critical coping mechanisms for women experiencing abortion, but they are often ignored and undervalued in practice. In addition, there is limited research into the effect of spirituality and social support on psychological pain (Dangel & Webb, 2017), and it is noteworthy that there are no studies conducted with women getting an abortion. Assessing the spiritual needs and social support levels of women experiencing abortion contributes to providing individualized and holistic care that reduces the effects of psychological pain (Sinthuchai et al., 2022; Dangel and Webb, 2017). In this regard, it is thought that this study will contribute to the literature and guide healthcare professionals in reducing psychological pain and preventing mental disorders in women experiencing abortion.

### Objectives

The objectives of the current study were:

1. To determine the psychological pain, spiritual well-being and social support levels of women undergoing therapeutic or elective abortion,
2. To explore the relationship between psychological pain, spiritual well-being, and social support in women undergoing therapeutic or elective abortion,
3. To identify significant factors predicting psychological pain levels of women undergoing therapeutic or elective abortion.

## Methods

### Design

The cross-sectional study was conducted in the gynecology and obstetrics service of a city hospital in Turkey between March 2021 and December 2022.

### Sample and Participants

The population of the study consisted of women hospitalized in the gynecology and obstetrics service of a city hospital with a diagnosis of therapeutic or elective abortion. The sample size was calculated using the G^*^power 3.1.9.7 statistical software program (Faul et al., 2007). To determine the relationship between measurements, the minimum sample size was determined as 258 people according to α: 0.05, β: 0.10 and effect size: 0.20.Accordingly, 342 women who undergone therapeutic or elective abortion on the specified dates, and met the inclusion criteria were included in the study. The inclusion criteria were as follows: having undergone therapeutic or elective abortion, being married, being ≥ 18 years, being literate, agreeing to participate in the study. The exclusion criteria were as follows: having a history of recurrent miscarriage or infertility, having been diagnosed with any chronic/severe physical illness (diabetes mellitus, cardiac or respiratory diseases, etc.) or mental disorder (intellectual disability, somatic symptom disorder, depression, psychotic disorders, substance use disorder, etc.). After the study was completed, post-hoc power analysis was performed with the G^*^power 3.1.9.7 statistical software program. As a result of the power analysis, based on α: 0.05 and n: 342 people, the power level for determining the relationship between psychological pain and spiritual well-being (r: − 0.492) was found to be 1.00, and the power level for determining the relationship between psychological pain and social support (r: − 0.543) was found to be 1.

### Measures

Study data were collected with a personal information form, the psychological scale, spiritual well-being scale and multidimensional scale of perceived social support.

Personal Information Form: This form included 19 items about women’ socio-demographic (age, education level, duration of marriage, family type, employment status, economic level, etc.) and obstetrics characteristics (history of abortion, number ofabortion, current abortion type, cause of abortion, problems after abortion, etc.).

Psychache Scale (PS): PS is a self-report scale developed by Patterson and Holden, (2012) to assess psychological pain. The scale consists of 13 items, which are scored on a 5-point Likert-type scale from 1 (never or strongly disagree) to 5 (always or strongly agree). The highest and lowest scores on the scale are 65 and 13, respectively. Higher scores indicate more intense and frequent perceptions of psychological pain. A validity and reliability study on the adaptation of the scale into Turkish was performed by Demirkol et al. (2018), and Cronbach’s alpha coefficient was found to be 0.98 (Demirkol et al., 2018). Cronbach’s alpha value obtained in the present study was 0.94.

Spiritual well-being scale (SWBS): The SWBS was developed by Eksi and Kardas (2017) to determine the process of understanding and living people’s lives with their personal, social, environmental and transcendental aspects in line with their values and ultimate meanings. In the Turkish validity and reliability study of the scale, the Cronbach’s alpha coefficient was determined as 0.88. The SWBS consists of 29 items, each of which is scored on a five-point Likert-type scale with options ranging from 1 (not applicable to me at all) to 5 (completely applicable to me), and has three subscales, namely transcendence, harmony with nature and anomie. Total scores on the scale range between 29 and 145, and higher score signifies greater spiritual well-being (Eksi & Kardas, 2017). In the present study, Cronbach’s alpha coefficient was calculated as 0.84for the total scale.

Multidimensional Scale of Perceived Social Support (MSPSS): This scale was developed by Zimet et al. (1988) to determine the social support characteristics perceived by individuals. The Turkish validity and reliability study was conducted by Eker et al. (2001), and the reliability coefficient was found to be 0.89. The scale consists of 12 seven-point Likert-type items, and each item is scored as 1 (very strongly disagree) to 7 (very strongly agree). The MSPSS has three subscales as family, friends and significant other. The total score that can be obtained from the scale varies between 12 and 84. High scores indicate high perceived social support (Eker et al., 2001). Cronbach’s alpha value of the MSPSS was found to be 0.87 in this study.

### Data Collection

The women in this study were informed about the study, and written consent was obtained from those who met the inclusion criteria. The data were collected by the researcher via the face-to-face interview method. Each interview took about 30–40 min. Pandemic-related measures, such as masks, social distance and hygiene were followed during the data collection.

### Ethical Considerations

This study was approved by the Bozok University Clinical Research Ethics Committee (Decision date and no: 2019.04.17–11). Before the data collection process was initiated, the institutional permission from the chief of Yozgat City Hospital was obtained. In addition, the principle of volunteerism was adopted in the study, and women’ written consent was obtained.

### Data Analysis

Data were analyzed on the SPSS 22.0 software (SPSS, Inc., Chicago, IL, USA). The normality of the data was assessed by the Kolmogorov–Smirnov test. Categorical data were expressed as n (%), and the ratio data were described as mean ± SD. The independent samples *t*-test or one-way analysis of variance (Bonferroni test as a post-hoc comparison) were used to compare the psychological pain scores according to women’ socio-demographic and obstetrics characteristics. Pearson’s correlation analysis was used to examine the relationship between psychological pain, spiritual well-being, and social support. The effects of all variables on psychological pain were examined using multiple linear regression analysis (backward elimination method). The multicollinearity test was employed to decide which variables to include in the model. Variables with a variance inflation factor value of < 10, a tolerance value of > 0.2, and a condition index value of < 15 were included in the model. The explanatory power of the regression model was evaluated with Adjusted R square (Adj. R^2^). The significance level was accepted as *p* < 0.05.

## Results

### Women’ Characteristics

Of the women who got an abortion, 60.2% were aged 18–30 years, 36.3% were high school graduates, 64.0% were not employed, 56.7% had a middle economic level, 88.9% lived in a nuclear family, 86.8% were non-smokers, and none of them used alcohol. According to the women’s statements, 62.3% got married at the age of 18–24, 69.0% had been married for ≤ 10 years, 32.7% had ≥ 3 pregnancies before, 43.0% had one child, and 39.3% used an intrauterine device as a family planning method. Additionally, 35.1% had had an abortion before, 16.7% had had an abortion ≥ 2 times before, the current abortion of 53.2% was for therapeutic reasons, 40.5% had an abortion in the 9th-12th week of their pregnancy, the reason for the abortion was unplanned pregnancy in 46.8%, and 53.2% reported that they had problems after abortion.

### Women’ Psychological Pain, Spiritual Well-Being and Social Support Levels

In the present study, the mean scores of women, who had undergone therapeutic or elective abortion, were 26.96 ± 11.21 on the PS, 110.72 ± 13.09 on the total SWBS, and 64.09 ± 15.62 on the total MSPSS (Table 1).

**Table 1:** Mean scores of the PS, SWBS and MSPSS

Scales	Mean ± SD	Min–Max	Range
Psychache scale	26.96 ± 11.21	13–55	13–65
Spiritual well-being scale	110.72 ± 13.09	67–144	29–145
Transcendence	61.37 ± 8.12	25–75	15–75
Harmony with nature	28.39 ± 4.33	12–35	7–35
Anomie	20.95 ± 6.46	7–35	7–35
Multidimensional scale of perceived social support	64.09 ± 15.62	12–84	12–84
Family	21.57 ± 5.40	4–28	4–28
Friends	20.85 ± 5.47	4–28	4–28
Significant other	21.62 ± 5.72	4–28	4–28

*PS* Psychache scale, *SWBS* spiritual well-being scale, *MSPSS* multidimensional scale of perceived social support

### Psychological Pain Levels by Some of Women Characteristics

Table 2 shows the comparison of women’s psychological pain levels in terms of some characteristics. Accordingly, psychological pain levels were significantly higher in women who were aged 31–48 years, were primary school graduates, were unemployed, lived in an extended family with poor economic status, had been married for ≥ 11 years, had been pregnant ≥ 3 times before, had ≥ 2 children, had a history of abortion, had had ≥ 2 abortions before, underwent elective abortion now, had an abortion in the ≥ 13th week of gestation, had an abortion due to unplanned pregnancy, and had post-abortion problems (*p* < 0.05).

**Table 2:** Women’ characteristics and comparison of psychological pain

Characteristics	n	%	Mean ± SD	t/F	*p*
*Age (Mean ± SD: 29.44 ± 5.46)*					
18–30 years	206	60.2	25.49 ± 9.45	3.023^*^	0.003
31–48 years	136	39.8	29.20 ± 12.19		
*Education level*					
Primary school	110	32.2	30.27 ± 13.51	9.586^**^	< 0.001
High school	124	36.3	26.80 ± 10.06		
University	108	31.5	23.78 ± 8.78		
*Employment status*					
Employed	123	36.0	22.45 ± 8.25	6.456^*^	< 0.001
Unemployed	219	64.0	29.51 ± 11.86		
*Economic level*					
Bad	66	19.3	33.67 ± 15.05	20.901^**^	< 0.001
Moderate	194	56.7	26.63 ± 9.43		
Good	82	24.0	22.37 ± 8.84		
*Family type*					
Nuclear family	304	88.9	26.45 ± 11.10	2.445^*^	0.015
Extended family	38	11.1	31.14 ± 11.37		
Smoking status					
Smoking	25	7.4	28.48 ± 10.69	0.474^**^	0.623
Not smoking	297	86.8	25.21 ± 13.11		
Quit smoking	20	5.8	26.95 ± 11.15		
*Marriage age (Mean ± SD: 21.10 ± 3.69)*					
< 18 years	80	17.5	27.76 ± 10.97	0.150^**^	0.861
18–24 years	213	62.3	26.77 ± 13.51		
≥ 25 years	69	20.2	26.39 ± 9.84		
*Duration of marriage (Mean ± SD: 8.37 ± 4.20)*					
≤ 10 years	236	69.0	25.88 ± 9.43	2.675^*^	0.008
≥ 11 years	106	31.0	29.37 ± 12.17		
*Previous pregnancies (Mean ± SD: 2.00 ± 1.54)*					
No pregnancy	64	18.7	23.91 ± 7.89	6.957^**^	< 0.001
1 pregnancy	69	20.2	25.28 ± 10.70		
2 pregnancy	97	28.4	27.08 ± 10.93		
≥ 3 pregnancy	112	32.7	30.51 ± 12.45		
*Number of children (Mean ± SD: 2.89 ± 0.81)*					
No children	64	18.7	24.62 ± 10.07	4.545^**^	0.011
1 children	147	43.0	27.50 ± 11.16		
≥ 2 children	131	38.3	29.13 ± 12.44		
*Family planning method*					
Not using	61	17.8	29.06 ± 10.19	2.619^**^	0.051
Condom	99	28.9	26.86 ± 11.20		
Intrauterine device	134	39.3	23.86 ± 10.74		
Withdrawal	48	14.0	27.62 ± 11.77		
*History of abortion*					
No	222	64.9	25.82 ± 10.65	2.596^*^	0.010
Yes	120	35.1	29.09 ± 11.96		
*Number of abortion*					
No abortion	222	64.9	25.94 ± 10.65	3.783^**^	0.024
1 abortion	63	18.4	27.26 ± 10.82		
≥ 2 abortion	57	16.7	30.31 ± 12.90		
*Current abortion type*					
Therapeutic abortion	182	53.2	25.12 ± 10.41	3.294^*^	0.001
Elective abortion	160	46.8	29.06 ± 11.76		
*Gestational week (Mean ± SD: 10.52 ± 3.58)*					
2–8 weeks	121	35.4	24.95 ± 10.01	13.095^**^	< 0.001
9–12 weeks	138	40.5	25.64 ± 10.23		
≥ 13 weeks	83	24.3	32.24 ± 13.07		
*Cause of abortion*					
Unplanned pregnancy	160	46.8	28.89 ± 11.70	5.004^**^	0.007
Intrauterine fetal loss	94	27.5	24.46 ± 10.29		
Bleeding	88	25.7	26.15 ± 10.75		
*Problems after abortion*					
No	131	46.8	25.27 ± 11.36	2.206^*^	0.027
Yes	211	53.2	28.02 ± 11.03		

*Independent samples *t*-test; **One-way analysis of variance

### Correlation Between Psychological Pain, Spiritual Well-Being, and Social Support

The correlation between between psychological pain, spiritual well-being, and social support was shown in Table 3. Pearson’s correlation analysis revealed a negative correlation between PS and SWBS (r = 0.492, *p* < 0.001), and MSPSS (r = 0.543, *p* < 0.001).

**Table 3:** The Correlation between PS, SWBS, and MSPSS

Scales		SWBS	MSPSS
Psychache Scale	r	− 0.492	− 0.543
	*p*	< 0.001^**^	< 0.001^**^

*PS* psychache scale, *SWBS* spiritual well-being scale, *MSPSS* multidimensional scale of perceived social support

**p < 0.01

### Factors Predicting Women’ Psychological Pain Levels.

The results of the multivariate linear regression analysis showing the factors that affected women’ psychological pain levels are given in Table 4. The potential predictors showing statistically significant association with the *t*-test, ANOVA or correlation test were selected in the regression analyses. The predictive power of the linear regression model calculated using the backward elimination method (Adjusted R^2^) was 44.9%. Significant predictors of psychological pain levels of the Turkish women undergoing therapeutic or elective abortion were spiritual well-being (β = 0.105), social support (β = 0.451), age (β = 0.122), employment status (β = 0.151), economic level (β = 0.214), history of abortion (β = 0.217), number of abortion (β = 0.251), current abortion type (β = 0.106), and gestational week (β = 0.129) (*p* < 0.05).

**Table 4:** Predictive factors of women’ psychological pain

Variables	B	SE	β	t	*p*	95% CI LL	UL
Constant	40.241	6.705		6.002	< .001	27.051	53.431
Spiritual well-being (continuous)	− 0.090	0.038	− 0.105	− 2.370	0.018	− 0.164	− 0.015
Social support (continuous)	− 0.324	0.032	− 0.451	− 10.273	< 0.001	− 0.386	− 0.262
Age (continuous)	0.251	0.089	0.122	2.822	0.005	0.076	0.427
Employment status	3.532	1.059	0.151	3.336	0.001	1.449	5.614
Economic level	− 3.658	0.724	− 0.214	− 5.049	< 0.001	− 5.614	− 2.233
History of abortion	5.086	2.178	0.217	2.336	0.020	0.802	9.369
Number of abortion (continuous)	3.689	1.356	0.251	2.721	0.007	1.022	6.356
Current abortion type	2.390	0.944	0.106	2.531	0.012	0.532	4.247
Gestational week (continuous)	0.405	0.130	0.129	3.104	0.002	0.148	0.661

R: 0.670, Adj. R2: 0.449, F: 30.004, *p:* < 0.001

Adj. R2: Adjusted R square; B: Partial regression coefficient; β: Standard partial regression coefficient; 95% CI: 95% confidence interval

Reference categories: 1; Variables: employment status: 1 employed; economic level: 1 bad; history of abortion: 1 no; current abortion type: 1 therapeutic abortion

## Discussion

Abortion, one of the common complications during pregnancy, is a traumatic process that affects both the physical and psychological health of women (Traylor et al., 2020). In addition, pregnancy loss is considered an experience that contributes to the development of psychological pain (Crockett et al., 2021). However, in the literature, the concept of psychological pain has generally been studied in homeless people (Patterson & Holden, 2012), depressive patients (Yeşiloğlu et al., 2023), prisoners (Pereira et al., 2010), and students (Wang et al., 2023), but there is no study with women experiencing abortion. In this regard, it is anticipated that the current research conducted to determine psychological pain and related factors in women experiencing abortion will provide evidence for planning individualized psychosocial-spiritual care and counseling services for women. In this study, the mean psychological pain score was determined as 26.96 ± 11.21. Based on this, it can be said that the psychological pain levels of women who got an abortion were low, but the lack of studies with this group makes it difficult to evaluate the results. In studies conducted with different groups in the literature, the mean psychological pain score was determined as 39.98 ± 12.96 in patients with depression (Tanriverdi et al., 2022), 37.6 ± 14.28 in patients with schizophrenia (Demirkol et al., 2019), and 37.6 ± 15.3 in patients with obsessive–compulsive disorder (Demirkol et al., 2019). The lower psychological pain in women who got an abortion may have been because the aforementioned studies (Demirkol et al., 2019; Demirkol et al., 2019; Tanrıverdi et al., 2022) had been conducted with individuals with mental disorders. For this reason, it is thought that more research is needed to more clearly understand the psychological pain experienced by women getting an abortion.

In the current study, the mean spiritual well-being score of women, who got an abortion, was 110.72 ± 13.09. In a different study conducted with pregnant women in Türkiye, it was determined that the spiritual well-being levels of pregnant women were high (125.59 ± 12.97) (Bilgiç & Çıtak Bilgin, 2021). In a study conducted in Iran, it was found that pregnant women had a moderate level of spiritual well-being (Abdollahpour & Khosravi, 2018). Spirituality is defined as “one’s striving for and experience of a connection with the essence of life” (de Jager Meezenbroek et al., 2012). In this study, which was conducted in Türkiye, a Muslim country, women’s high spiritual well-being is pleasing, as it supports them in coping with their losses. However, studies in the literature on women getting a therapeutic or elective abortion are generally inadequate in number (Alipanahpour et al., 2023). In fact, determining the problems experienced by these women and the associated risk or protective factors is essential for the protection and development of women’s mental health (Şimşek, 2022).

The social support system, which contributes to coping with stressful life events, is considered a powerful resource for the prevention, solution, and treatment of the individual’s sociological and psychological problems (Iwanowicz-Palus et al., 2021). In this study, the mean social support score of women who got an abortion was 64.09 ± 15.62, and it can be said that the level of social support they perceived was good. In different studies conducted using the same measurement tool, it was reported that the social support perceived by women who experienced a pregnancy loss was at a medium level (Akdag Topal & Terzioglu, 2019; Palas Karaca & Oskay, 2021). Based on this, it can be said that the level of social support in women experiencing abortion varies. This variability may be due to the difference in the sociocultural characteristics of the sample group.

In this study, it was determined that both spiritual well-being and perceived social support were significant variables affecting psychological pain. Similarly, previous studies, although they were not conducted with women getting an abortion, indicated that spirituality was effective in reducing psychological pain along with social support (Masik et al., 2022; Zhang et al., 2022). The existing social support network in people’s lives and spiritual well-being provides a protective factor against the risks of psychological pain. The decrease in protective factors such as social support and spiritual well-being increases the sensitivity to the risk of psychological pain (Dangel & Webb, 2017). In this regard, it becomes clear that the concepts of spirituality and social support should be taken into consideration to reduce psychological pain experienced by women getting an abortion.

When the socio-demographic predictors of psychological pain in women getting an abortion were examined in depth, it was determined that age was an important factor. As women get older, their psychological pain levels also increase. In studies on the relationship between abortion and anxiety in the literature, it was determined that women’s anxiety about loss or abortion increased as age increased (Gao et al., 2020; Gümüşssoy et al., 2021). On the other hand, in the current study, women who did not have a job and had poor economic status were found to experience more psychological pain. It is thought that several socioeconomic factors, such as low income, financial problems and concerns, and economic dependency may have an impact on the concept of psychological pain (Wang et al., 2016).

The type of abortion, which is one of the obstetric characteristics, is also a variable affecting psychological pain. In this study, the psychological pain levels of women who experienced elective abortion were found to be higher than the levels of those who experienced therapeutic abortion. Türkiye is a country where people who believe in Islam are in the majority. According to Islam, the curettage of a baby without any risk is a sin because it is ending a life (Şimşek Çetinkaya & Şimşek, 2023; Masik et al., 2022). In the current study, this situation is thought to be effective in the high psychological pain levels of women who got an elective abortion. At the same time, those with higher gestational weeks had higher levels of psychological pain. This result may be related to the increase in the level of mother-infant attachment in parallel with the increase in gestational age (Oreg, 2020). In this study, the history of abortion and number of abortions were other variables that affected psychological pain. The likelihood of not having a healthy child may have increased psychological pain in women who had a history of previous abortion and who had experienced more than one (Onaolapo et al., 2020). Accordingly, it can be emphasized that the number of studies on determining psychological pain and related factors in women experiencing abortion should be increased.

### Strengths and Limitations

This study is valuable in that it is the first research on the investigation of psychological pain, spiritual well-being, and social support in women undergoing abortion. It is thought that the results will guide healthcare professionals in protecting the mental health of women experiencing abortion. However, this study has several limitations. First, due to cultural or religious differences, the results are specific only to women experiencing abortion in Turkish Muslim society. Second, since the study was conducted only in one hospital, it cannot be generalized to all women experiencing abortion. Third, the current study was cross-sectional, and therefore a causal relationship could not be established. That’s why, studies with large samples can be conducted to examine variables related to and causal relationships between psychological pain, spiritual well-being, and social support in women experiencing abortion.

## Conclusion

In this study, the psychological pain level of women who experienced abortion was determined to be low, and their spiritual well-being and social support levels were high. The results of this study show that psychological pain, spiritual well-being, and social support are interrelated dynamics. In addition, the concept of psychological pain is affected by socio-demographic and obstetric factors, such as age, employment status, economic level, history of abortion, number of abortions, current abortion type and gestational week, apart from spiritual well-being and social support. All these variables explained 44.9% of the psychological pain in Turkish women undergoing abortion.

### Implications for Clinical Practice

The results of this study allow some conclusions at the clinical level implication. In this regard, it is of great importance for health professionals serving women who have experienced abortion to evaluate them in terms of psychological pain, and to determine their spiritual needs and social support sources. In addition, taking into account the socio-demographic and obstetrics characteristics of women undergoing abortion, interventions, such as biopsychosocial care, psychoeducation, psychotherapy, spiritual counseling, support groups can be planned with a multidisciplinary team to reduce psychological pain. Additionally, mixed-design studies with larger samples can be conducted to better understand the variables affecting the psychological pain of women experiencing abortion.

## Data Availability

The data of this study are available from the corresponding author upon reasonable request.

## Appendix 1. Spiritual Well-Being Scale

**Table:**

1	Being connected to a divine power gives me confidence
2	I think that nature should be respected
3	I feel a sense of dissatisfaction with life
4	When I encounter a problem, I feel God's help
5	I believe that God knows all my secret and oRpen feelings and thoughts
6	I think that all living things deserve respect
7	There is a big void in my life
8	I witness the power of God in my daily life
9	I believe that God loves and cares about me
10	I treat all living things on earth well
11	I don't enjoy life
12	I feel the presence of God at every moment of my life
13	The feeling of taking refuge in a stronger being comforts me
14	I see myself as a part of nature
15	I still haven't found the purpose of my life
16	I believe that there is good in every event that happens to me
17	My faith guides me on how to live my life
18	The rights of all living things on earth are important to me
19	I don't know where to begin to solve my problems
20	When I am alone, I think about Allah and his creations
21	My beliefs and values increase my ability to endure difficulties
22	I live in harmony with nature
23	I feel overwhelmed when I experience challenges
24	My faith allows me to see that there can be positive aspects even in the troubles I experience
25	Nothing in life happens without a reason
26	I think that life consists of events that make me unhappy
27	Knowing that not everything is in my hands is a source of consolation in the face of events that upset me
28	I believe that every natural entity on earth is unique
29	Believing that worldly life is temporary purifies me from my ambitions
